# PREDICTORS OF SELF-CONTINUITY IN LATER LIFE

**DOI:** 10.1093/geroni/igz038.1128

**Published:** 2019-11-08

**Authors:** Charikleia Lampraki, Dario Spini, Daniela Jopp

**Affiliations:** 1 University of Lausanne, Swiss National Centre of Competence in Research LIVES, Lausanne, Vaud, Switzerland; 2 University of Lausanne, Lausanne, Switzerland, Switzerland

## Abstract

Self-continuity is an identity mechanism that inter-connects past and present experiences with future expectations, creating a coherent whole. However, research is limited regarding inter-individual differences and life course determinants of change in self-continuity. Using a life-course perspective on vulnerability, we investigate how the accumulation of resources (e.g., social, hopeful attitude) and the occurrence of critical life events (e.g., childhood adversity, divorce) across the life course may affect changes in self-continuity. Data derived from the LIVES Intimate Partner Loss Study conducted in Switzerland from 2012 to 2016 (3 waves). The sample consisted of individuals having experienced divorce (N = 403, Mage = 55.43) or bereavement (N = 295, Mage = 69.91) in the second half of life, using a continuously married group as a reference (N = 535, Mage = 65.60). Multilevel hierarchical models were used. Results indicated that as individuals grew older they experienced more self-continuity. More childhood adversity was negatively associated with inter-individual differences in self-continuity for all groups. Divorcees with more childhood adverse events felt significantly less self-continuity as they grew older than divorcees with less childhood adversity. In the bereaved group, more childhood adversity and less hope was linked to lower levels of self-continuity. More hopeful married individuals felt more self-continuity as they grew older than less hopeful ones. In sum, findings demonstrate that self-continuity changes as a function of age, but also differs with regard to the critical life events experienced across the life course and the availability of resources.

